# A p-i-n junction diode based on locally doped carbon nanotube network

**DOI:** 10.1038/srep23319

**Published:** 2016-03-21

**Authors:** Xiaodong Liu, Changxin Chen, Liangming Wei, Nantao Hu, Chuanjuan Song, Chenghao Liao, Rong He, Xusheng Dong, Ying Wang, Qinran Liu, Yafei Zhang

**Affiliations:** 1 Key Laboratory for Thin Film and Microfabrication of the Ministry of Education, National Key Laboratory of Science and Technology on Micro/Nano Fabrication, Department of Micro/Nano Electronics, School of Electronic Information and Electrical Engineering, Shanghai Jiao Tong University, Shanghai, China

## Abstract

A p-i-n junction diode constructed by the locally doped network of single-walled carbon nanotubes (SWNTs) was investigated. In this diode, the two opposite ends of the SWNT-network channel were selectively doped by triethyloxonium hexachloroantimonate (OA) and polyethylenimine (PEI) to obtain the air-stable p- and n-type SWNTs respectively while the central area of the SWNT-network remained intrinsic state, resulting in the formation of a p-i-n junction with a strong built-in electronic field in the SWNTs. The results showed that the forward current and the rectification ratio of the diode increased as the doping degree increased. The forward current of the device could also be increased by decreasing the channel length. A high-performance p-i-n junction diode with a high rectification ratio (~10^4^), large forward current (~12.2 μA) and low reverse saturated current (~1.8 nA) was achieved with the OA and PEI doping time of 5 h and 18 h for a channel length of ~6 μm.

The p-n junction diode is the building block of integrated circuits and photovoltaic devices. The semiconducting single-walled carbon nanotube (SWNT) is an ideal candidate for constructing the diode owing to its unique one-dimensional (1D) structure and extraordinary electrical properties^1^. The doping type and degree of the SWNT can be tuned by non-covalent doping without introducing structure defects^2^,^3^. The energy gap of the SWNT can be controlled by its diameter to fabricate the p-n junction diodes with different characteristics^4^. Moreover, SWNTs possess a high intrinsic mobility (>100,000 cm^2^/Vs)^5^,^6^ and a large current-carrying capacity^7^,^8^ up to 10^9^ A cm^−2^.These characteristics make SWNTs promising for use in fabricating high-performance diodes.

Some types of SWNT diodes had been investigated in previous reports^9^,^10^. A split-gate geometry was used to fabricate the p-n diode^11^,^12^,^13^. The split gates were used to electrostatically dope the SWNT and thereby caused formation of a p-n junction. However, the processes of this device were complex and needed to fabricate the buried metals as the split gate electrode. Another structure based on asymmetric metal/nanotube contacts was also used to fabricate the diode^4^,^14^,^15^. In this device, the SWNT was contacted with high and low work function metal to form the p- and n-type Schottky barrier respectively at the contacts, resulting in built-in electric field in the SWNT. But, this kind of diode required an extra gate terminal to realize the best rectification performance. In addition, the use of individual or sparse SWNT array in those devices also limited the forward output current^16^,^17^,^18^,^19^. Therefore, good device design and fabrication technology were highly desired to achieve high-performance SWNT diodes.

A p-i-n junction diode based on the selected-area doping of the SWNT network was therefore investigated in this study. In this diode, the two areas in the opposite ends of the SWNT network channel were selectively doped by triethyloxonium hexachloroantimonate (OA) and polyethylenimine (PEI) to form the air-stable p-type and n-type regions, respectively. Meanwhile, the middle of the SWNT network remained intrinsic, causing the formation of a p-i-n junction with a strong built-in electric field in the SWNT network. The results show that the forward current and rectification ratio increased as the doping degree increased, and the forward current could be increased by decreasing the channel length. A high-performance diode with a high rectification ratio (~10^4^), large forward current (12.2 μA), and low reverse-saturated current (1.8 nA) was achieved with OA and PEI doping times of 5 h and 18 h, respectively, and a channel length of ~6 μm.

## Results

### Optical absorption characteristics of the doped SWNT network

**Figure 1:**
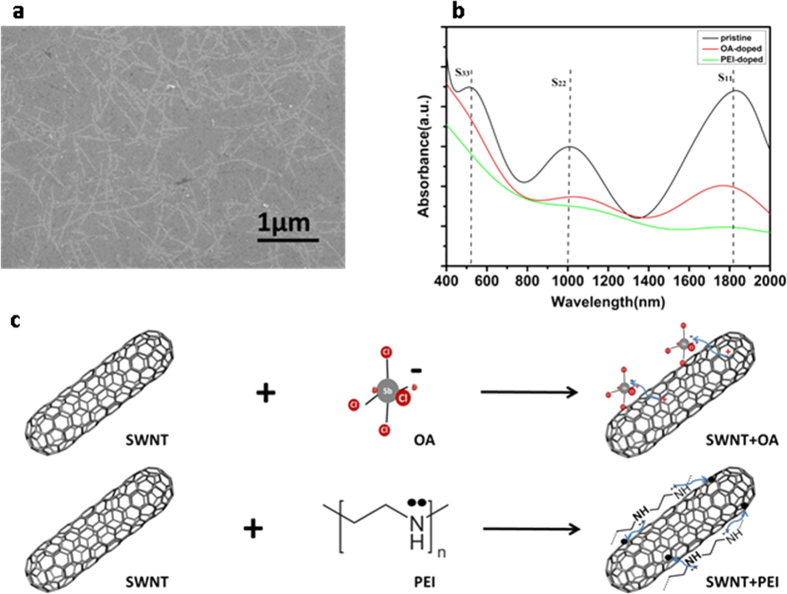
Assembled random network of carbon nanotubes and their optical absorption characteristics before and after doping. (**a**) A scanning electron microscope (SEM, Model Zeiss Ultra 55) image of assembled random network of carbon nanotubes. (**b**) UV-Vis-NIR spectra of pristine, OA-doped, and PEI-doped SWNT network. (**c**) Schematic diagram showing the doping process of OA and PEI to SWNTs, respectively. OA forms a charge-transfer complex with the SWNTs, injecting holes into the SWNT network. Electrons from amine groups are donated to the SWNT network when PEI winds around the carbon nanotube.

To observe the doping effect, optical absorption of the SWNT network was performed. The scanning electron micrograph (SEM) in Fig. 1 are presents the random distribution of the SWNT network (density of ~1.4 μm^−2^) on wafer fabricated using a self-assembly technique^20^ (detailed in the Methods section). Figure 1b shows the UV-Vis-NIR absorption spectra for the pristine, OA, and PEI doped SWNT network. Three main absorption bands, namely S_11_, S_22_, and S_33_, are clearly observed for the pristine SWNT network, which is attributed to the 1D band structure of semiconducting SWNTs. The S_11_ absorption band observed at ~1700–1900 nm corresponds to the first set of Van Hove singularity transitions. Similarly, the S_22_and S_33_ absorption bands observed at ~900–1200 nm and ~450–600 nm correspond to the second and third set of Van Hove singularity transitions, respectively. No M_11_ absorption peak is observed in the absorption spectrum, which indicates low metallic content of the SWNTs. As a single-electron oxidant^21^, shown in Fig. 1c, OA receives an electron from the valence band of the SWNTs, resulting in p-type SWNTs. The attenuation of absorption peaks occur on account of the depletion of electrons from the valence band of the SWNTs, which is evidence of the successful doping of OA. On the other hand, PEI provides electrons to the conduction band of SWNTs, resulting in n-type SWNTs^3^. Similar behaviour of absorption peak attenuation is observed for PEI doping on account of the increased number of electrons in the conduction band.

### Characteristics of field-effect transistors (FETs) with entire SWNT channel doped

**Figure 2:**
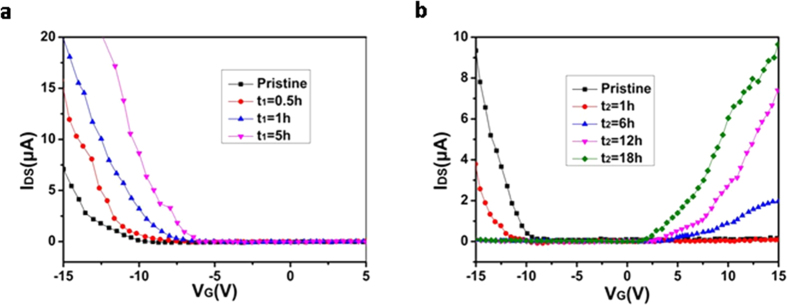
Transfer characteristics of a SWNT-FET before and after doping. (**a**) Transfer characteristics of an SWNT-FET at V_DS_ = 1.5 V before and after OA doping with a doping time (t_1_) of 0.5 h, 1 h, and 5 h, respectively. (**b**) Transfer characteristics of an SWNT-FET at V_DS_ = 1.5 V before and after PEI doping with a doping time (t_2_) of 1 h, 6 h, and 12 h, respectively.

To further study the doping feature, we fabricated SWNT network transistors (SWNT-FET) on SiO_2_/Si substrate by evaporating 30-nm Au as the source and drain electrodes. The electrical characteristics of SWNT-FET altered after OA (Fig. 2a) and PEI (Fig. 2b) doping under ambient conditions. Prior to doping, stepping of the gate voltage (V_g_) towards the positive value decreases the conductance in pristine SWNTs, thereby indicating p-type FET characteristics. This p-type behaviour is observed on account of the oxidation of the FET device in an ambient environment^22^,^23^. With OA doping, the behaviour of curves dramatically changes and current drawn from drain to source (I_DS_) increases, as shown in Fig. 2a. The OA doping increases the hole concentration within SWNTs; thus, a smaller negative Vg is needed to open the device.

The p-type FET was converted into the n-type FET after PEI doping (Fig. 2b). The conductivity through the FET increased as V_g_ moves toward more positive values, which is behaviour that is completely antithetical to that of the OA-doped devices. The behaviour of FET devices was tuned by controlling the doping time.

### Characteristics of p-i-n diodes with the selected SWNT channel doped

**Figure 3:**
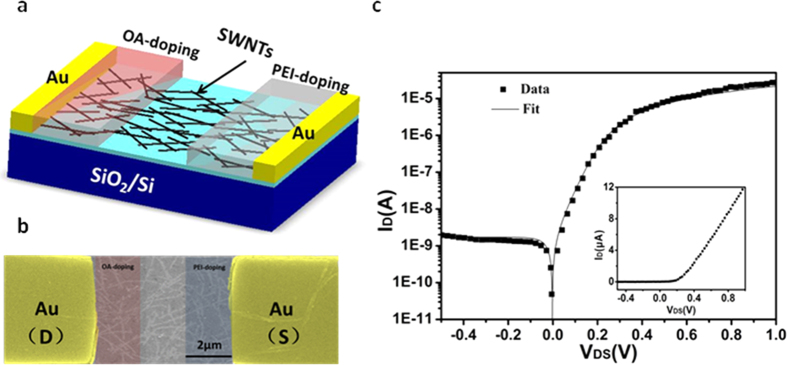
Structure and dark I_DS_-V_DS_ characteristics of the p-i-n diode. (**a**) Schematic diagram and (**b**) SEM image of the p-i-n diode. The density of this SWNT network is ~ 1.4 μm^−2^. The channel length is 6 μm and is evenly divided into three parts, as indicated by the arrows. The width is 5 μm. (**c**) Logarithmic plot of dark I–V characteristics of this SWNT network p-i-n diode at T = 300 K. The interval of each data point is 4 ms. Inset: Linear plot of I–V characteristics.

The p-i-n diodes were fabricated using a doping procedure similar to the one mentioned above. Selective areas were chosen on the different parts of the channel in a single device. To configure a SWNT network channel as a p-i-n diode, local doping was achieved by covering one end of the wafer using PMMA resists, while the other end is opened for doping using an e-beam lithography technique. One end of the channel thereby becomes the p-type (by OA doping; doping time set as t_1_), whereas the other end becomes the n-type (by PEI-doping; doping time set as t_2_). Figure 3a,b respectively show a schematic diagram of the device and a scanning electron micrograph of a p-i-n diode based on the SWNT network with a channel length of 6 μm. The p-type and n-type regions each have lengths of 2 μm doped by OA and PEI, respectively.

Figure 3c shows the I_DS_-V_DS_ characteristics of the p-i-n diode which was fabricated by locally doping SWNT channel with the OA and PEI for 5 h (t_1_) and18 h (t_2_) respectively. The curve shows good rectifying properties. As evident in the curve, when the device is forward-biased, the current (I_D_) rapidly increases up to 12.2 μA when biased at 1 V. Meanwhile, for reverse-biasing of the device, no effective current is observed. The characteristics in Fig. 3c can be fitted by the following equation^24^


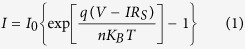


where I_0_ is the reverse bias leakage current, q is the unit charge, K_B_ is Boltzmann’s constant, T is absolute temperature, R_S_ is series resistance, and n is the ideality factor, takes on values between 1 and 2, depending on the nature of electron-hole generation and recombination mechanism. The diode parameters of I_0_ = 1.8 nA, R_S_ = 30 kΩ, and n = 1.3 were extracted by fitting at low bias condition. The rectification ratio for the device is as high as ~10^4^ when V_DS_ = ± 1 V.

**Figure 4:**
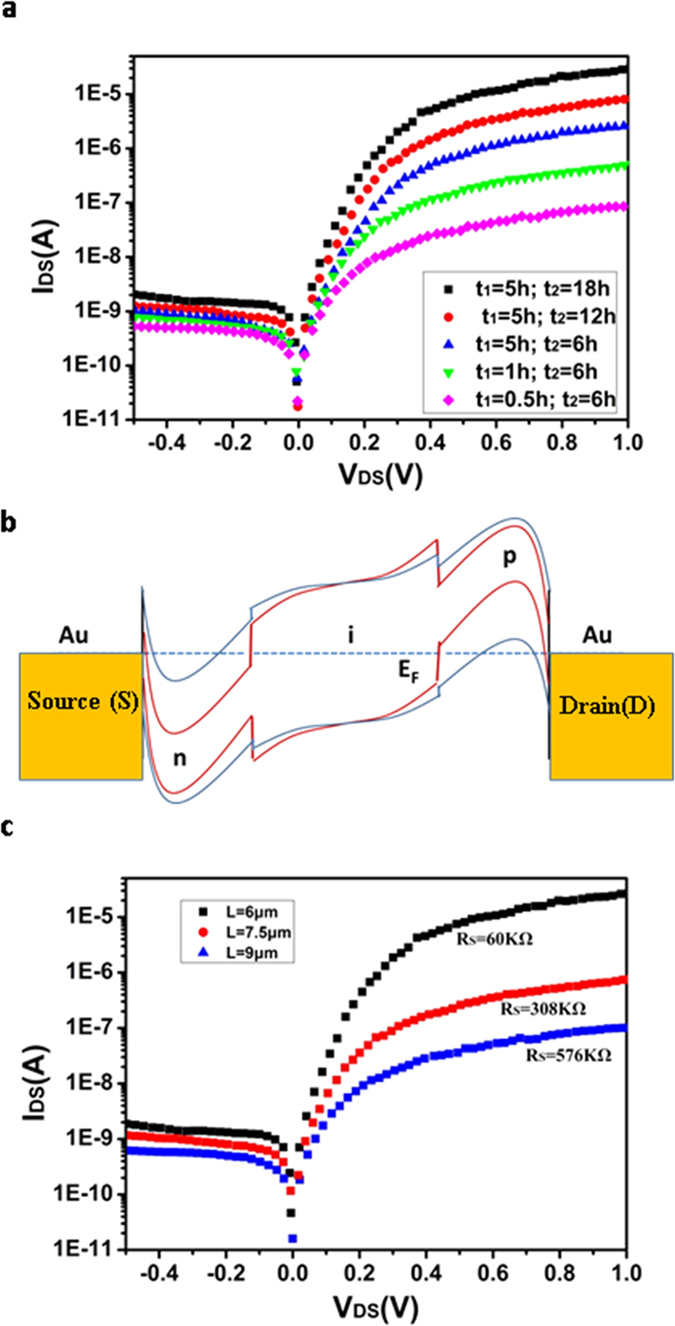
Dark I_DS_-V_DS_ characteristics of the SWNT network p-i-n diode with different doping degrees and different channel lengths. (**a**) Dark I_DS_-V_DS_ characteristics of the SWNT network p-i-n diode with different doping degrees. Its entire channel length is 6 μm. t_1_ is at a constant of 5 h, with t_2_ increasing from 6 to 18 h; t_2_ is at a constant of 6 h, with t_1_ increasing from 0.5 to 5 h. (**b**) Energy band diagram of the p-i-n device without bias. The red solid line denotes the reduction of the band gap at higher doping fractions. (**c**) I_DS_-V_DS_ characteristics of the SWNT network p-i-n diode with different channel lengths.

The effect of doping time on the electrical performance of the device was investigated. In the experiment, the same device was doped by the OA for 0.5 h, 1 h, and 5 h respectively when fixing the doping time of the PEI at 6 h. Then, the doping time of the PEI was increased to 12 h and 18 h respectively with the doping time of OA fixed at 5 h. These devices all showed diode characteristics. As shown in Fig. 4a, with the increase of the doping time, the forward current of the device increase from 96 nA to 12.2 μA and the rectification ratio increase from ~10^2^ to ~10^4^. Because the devices were fabricated on a silicon chip with thermally oxidated SiO_2_ insulation layer on the surface, there could be a small amount of trapped charges at the Si/SiO_2_ interface. The substrate of the devices was floated (no applied bias) when testing the device in our experiment. The trapped charges and the silicon substrate could cause additional doping in the SWNTs. However, it would not affect one to study the diode characteristics and get the best rectification performance of the diode by regulating the chemical doping of the SWNTs. Therefore, the effect of the trapped charges and silicon substrate are not considered here.

The increase of the device current under a higher doping degree in Fig. 4a can be understood by the band schematic of the device (Fig. 4b). The strong built-in electric field was formed between p region and n region after doping. The band bending at Au/SWNT interface is formed due to the Schottky contact between the SWNT and the metal electrodes. Previous studies indicated that the band gap of the SWNT would decrease with the increase of its doping level due to the band-gap renormalization (BGR) effect, which had been observed in the split-gate diode^24^,^25^,^26^. At a forward-bias state (a positive bias is applied to the drain), the barrier of the holes depends on the position of the valence-band top and the barrier of the electrons depends on the position of the conduction-band bottom. The valence-band top and the conduction-band bottom are higher and lower respectively when the doping degree of the SWNT is higher. Thus, both barriers for the holes and the electrons are smaller for the lower doping case, resulting in a larger device current.Similarly, at a reverse-bias state (a negative bias is applied to the drain), the barriers of the holes and the electrons are also lower for the lower doping case, causing a larger reverse device current. The increase of the rectification ratio with the increase of doping time in Fig. 4a can be attributed to a stronger built-in electric field formed at a higher doping case, as shown in Fig. 4b.

In addition, it is also important that the metal Au was selected as the contact in our device. Previous studies had shown that the Fermi level of the metal Au was located at slightly below the middle of the SWNT bandgap. Thus, the thin Schottky barriers could both be formed when the Au contacted with heavily-doped p-type and n-type SWNT, making the carriers be able to easily tunnel through the Schottky barrier and avoiding the high contact resistance.

The devices with variable channel lengths were also investigated. Fig. 4c shows results for three devices with channel lengths of 6 μm, 7.5 μm, and 9 μm, respectively. The channel width of 5 μm and the SWNT network density of ~1.4 μm^−2^ remain constant. Extending the channel length resulted in a larger series resistance (R_s_). The decrease of forward current was due to the increase of R_s_. It is worth mentioning that 6 μm is the optimal choice of channel length. Further channel length reduction leads to a short circuit caused by the existence of metallic carbon nanotubes.

## Discussion

In conclusion, we fabricated a p-i-n diode based on a selected-area chemical-doped SWNT network. The results show that the doping level can be effectively controlled by extending the doping time. The forward current and rectification ratio increase when increasing the doping degree of the SWNT network. This result can be due to a stronger built-in electric field. The series resistance (R_s_) can be modulated by varying the channel length. A diode with a high rectification ratio (~10^4^), large forward current (12.2 μA), and low reverse-saturated current (1.8 nA) was achieved when maintaining the doping time of OA and PEI at 5 h and 18 h, respectively, for the device with a channel length of ~6 μm. These results are a helpful contribution to theoretical research on the SWNT network p-i-n diode and are useful for the fabrication of SWNT devices.

## Materials and Methods

### Materials

The P3-SWNTs used in the present experiment were procured from Carbon Solutions Inc., US. The acidified SWNTs were cut to ~1 μm in length by way of ultrasonic treatment. Triethyloxonium hexachloroantimonate (OA) (Sigma-Aldrich) was dissolved in dichloroethane with a concentration of 5 mg/ml, and polyethylenemine (PEI, M_w_ 25000) (Sigma-Aldrich) was dissolved in methanol with a concentration of 10 wt.%.

### Assembly of the SWNT network

First, the degenerately 100-nm dry thermal SiO_2_-doped Si wafer was dipped in a mixture of 98% H_2_SO_4_ and 30% H_2_O_2_ (4:1v/v) at 350 K for 80 min to increase the surface hydrophilic characteristic. The Si wafer was again dipped in a 3-aminopropyltrimethoxysilane (APS) solution ((CH_3_)_2_CHOH: APS = 20:1v/v) for the subsequent 20 min accompanied by washing with DI water and drying under nitrogen atmosphere. Later, the treated Si wafer was subjected to suspension in 0.5 mg/ml P3-SWNTs aqueous solution at room temperature for 30 min. Finally, a randomly distributed SWNT network of a 1.4-μm^−2^density was obtained.

### Doping of SWNT network field effect transistor (FET)

For p-type doping, the SWNT-network FET was submerged in an OA solution for0.5 h, 1 h, and 5 h at 70 °C, followed by via rinsing with dichloroethane to remove excess dopant molecules. For n-type doping, the FET was submerged in PEI solution for 1 h, 6 h, 12 h, and 18 h followed by rinsing with methanol to remove nonspecifically adsorbed PEI.

### Fabrication of p-i-n diodes

Parallel Au electrode pairs were patterned on the assembled SWNT network using e-beam lithography (Denton Vacuum, EXPLOR-14) at a 100-nm thickness and with lift-off techniques. For improving the metal/SWNT contact, the devices were annealed at 200 °C in vacuum for 20 min. In the p-i-n junction diode, for the selective-area doping of the channel, a 300-nm thick layer of poly(methyl methacrylate) (PMMA) was spin-coated before maintaining the 5 μm × 2 μm window open in the PMMA resist at one end of the SWNT network channel using e-beam lithography. After that point, the wafer was maintained in the IPA solution for development for 20 s. An SWNT network channel of 2 μm within the window was exposed, while the other area on the chip was protected by the PMMA layer. Then, the resultant devices were immersed in the OA dopant solution for different time and followed by rinsing with dichloroethane to remove the excess OA. A similar process was repeated to open a window in the PMMA resist at selected opposite ends of the SWNT network channel. The devices were then immersed in the PEI dopant solution for different time and followed by rinsing with methanol to remove the excess PEI. Finally, the p-i-n diode was fabricated upon removal of the PMMA resist layer by rinsing the chip with acetone.

### Device characterization

The Agilent 4156C Precision Semiconductor Parameter Analyzer was used to perform the device characteristics. The substrate of the device was floated when measuring the device. All measurements were performed at room temperature.

## Additional Information

**How to cite this article**: Liu, X. *et al.* A p-i-n junction diode based on locally doped carbon nanotube network. *Sci. Rep.* **6**, 23319; doi: 10.1038/srep23319 (2016).
